# Interleukin-22 Plays a Protective Role by Regulating the JAK2-STAT3 Pathway to Improve Inflammation, Oxidative Stress, and Neuronal Apoptosis following Cerebral Ischemia-Reperfusion Injury

**DOI:** 10.1155/2021/6621296

**Published:** 2021-03-12

**Authors:** Yongfei Dong, Chengyun Hu, Chunxia Huang, Jie Gao, Wanxiang Niu, Di Wang, Yang Wang, Chaoshi Niu

**Affiliations:** ^1^Department of Neurosurgery, Anhui Provincial Hospital, Cheeloo College of Medicine, Shangdong University, Jinan, Shangdong, 250021, China; ^2^Department of Anesthesiology, The First Affiliated Hospital of USTC, Division of Life Sciences and Medicine, University of Science and Technology of China, Hefei, Anhui 230001, China; ^3^Department of Anesthesiology, The Second Affiliated Hospital of Anhui Medical University, Hefei, Anhui 230601, China; ^4^Department of Anesthesiology, The First Affiliated Hospital of Anhui Medical University, Hefei, Anhui 230022, China; ^5^Department of Neurosurgery, The First Affiliated Hospital of USTC, Division of Life Sciences and Medicine, University of Science and Technology of China, Hefei, Anhui 230001, China; ^6^Department of Neurosurgery, Anhui Provincial Hospital, Hefei, Anhui 230001, China

## Abstract

The interleukins (ILs) are a pluripotent cytokine family that have been reported to regulate ischemic stroke and cerebral ischemia/reperfusion (I/R) injury. IL-22 is a member of the IL-10 superfamily and plays important roles in tissue injury and repair. However, the effects of IL-22 on ischemic stroke and cerebral I/R injury remain unclear. In the current study, we provided direct evidence that IL-22 treatment decreased infarct size, neurological deficits, and brain water content in mice subjected to cerebral I/R injury. IL-22 treatment remarkably reduced the expression of inflammatory cytokines, including IL-1*β*, monocyte chemotactic protein- (MCP-) 1, and tumor necrosis factor- (TNF-) *α*, both in serum and the ischemic cerebral cortex. In addition, IL-22 treatment also decreased oxidative stress and neuronal apoptosis in mice after cerebral I/R injury. Moreover, IL-22 treatment significantly increased Janus tyrosine kinase (JAK) 2 and signal transducer and activator of transcription (STAT) 3 phosphorylation levels in mice and PC12 cells, and STAT3 knockdown abolished the IL-22-mediated neuroprotective function. These findings suggest that IL-22 might be exploited as a potential therapeutic agent for ischemic stroke and cerebral I/R injury.

## 1. Introduction

A report from the Global Burden of Disease (GBD) 2016 Stroke Collaborators showed that although the prevalence and mortality of stroke have decreased in the past 20 years, stroke remains the second leading cause of death and long-term disability worldwide [1, 2]. Among them, ischemic stroke is the most common type and occurs when cerebral arteries are occluded [3, 4]. Currently, restoring blood perfusion is an approved therapy for cerebral ischemic injury, including intravenous thrombolytic and endovascular therapy [5–8]. However, the degree of brain injury may be further aggravated following the reperfusion process, which is called cerebral ischemia/reperfusion (I/R) injury [9, 10]. Increasing evidence has shown that cerebral I/R can cause secondary brain injury, including cerebral hemorrhage, cerebral edema, and even death [11, 12]. Thus, it is necessary to clarify the pathological mechanism underlying cerebral I/R injury and explore novel therapeutic agents for ischemic stroke and cerebral I/R injury.

The interleukins (ILs) are a pluripotent cytokine family that have been reported to regulate ischemic stroke and cerebral I/R injury [13, 14]. In clinical experiments, higher IL-33 levels in acute ischemic stroke patients were positively correlated with better prognosis and could be used to predict outcomes and recurrences in acute ischemic stroke patients [15]. In addition, an IL-1 receptor antagonist (IL-1Ra) significantly decreased plasma concentrations of IL-6 and C-reactive protein in patients with ischemic stroke, indicating that IL-1Ra can improve clinical outcomes by reducing inflammation [16]. IL-35 pretreatment significantly reduced brain infarction and neurological deficits after cerebral I/R injury [17]. In addition, inhibition of IL-32 significantly reduced the infarct volume and neurological deficits following cerebral I/R injury by suppressing proinflammatory cytokine secretion [18].

IL-22 is a member of the IL-10 superfamily and plays important roles in tissue injury and repair [19, 20]. IL-22 has been reported to participate in various biological processes, including the inflammatory response, oxidative stress, endoplasmic reticulum stress, autophagy, apoptosis, and cell death [21–24]. Takahashi et al. reported that IL-22 treatment ameliorated I/R-induced myocardial injury and apoptosis by activating the signal transducer and activator of transcription (STAT) 3 signaling pathway [25]. Xu et al. also reported that IL-22 treatment or IL-22 overexpression prevented renal injury and inflammation after renal I/R in mice [26]. However, it remains unclear whether IL-22 is involved in ischemic stroke and cerebral I/R injury. Thus, the aim of this study was to determine the roles of IL-22 in cerebral I/R injury and to explore the underlying mechanism.

## 2. Materials and Methods

### 2.1. Animals and Animal Model

Male C57BL/6J mice were purchased from Beijing HFK Biotechnology Co., Ltd. (Beijing, China). All mice were maintained in standard housing conditions under a 12 h light-dark cycle and were allowed free access to standard rodent food and water. Animal care and procedures were conducted in accordance with the NIH *Guide for the Care and Use of Laboratory Animals* and approved by the Animal Ethics Committee of Anhui Medical University.

A middle cerebral artery occlusion (MCAO) model was generated according to previous research [27]. After 45 min of ischemia, the suture was removed to initiate reperfusion. The sham-operated mice underwent the same procedures but did not receive sutures. Thirty minutes before reperfusion, the mice were injected intraperitoneally with recombinant mouse IL-22 protein (rIL-22). The doses and times were selected according to our pilot experiments and previous research. The 90 mice were randomly allocated into the following three groups (*n* = 30/group): sham group, MCAO group, and rIL-22 group.

### 2.2. Neurological Impairment Scores

After 24 h of reperfusion, neurological impairment was evaluated as previously described [28]. The neurological scoring system ranged from 0 (no neurological deficits) to 4 (inability to walk spontaneously).

### 2.3. Measurement of Infarct Area

After neurological evaluation, the brains were rapidly removed and subsequently cut into coronal sections and then incubated with 2,3,5-triphenyltetrazolium chloride (TTC) at 37°C for 20 min. The sections were fixed in 4% paraformaldehyde and photographed using an HD camera. The infarct area was analyzed using ImageJ, and the infarct volume was calculated as previously described [29].

### 2.4. Brain Water Content

After 24 h of reperfusion, the mice were sacrificed and the brain tissues were rapidly removed and then immediately weighed to obtain the wet weight. Subsequently, the brain tissues were dried in a desiccating oven at 105°C to obtain the dry weight. The brain water content was calculated according to the previous described [30].

### 2.5. Cell Culture and Treatment

PC12 cells procured from the Culture Collection of the Chinese Academy of Science (Shanghai, China) were cultured in DMEM containing 10% fetal bovine serum and 1% penicillin/streptomycin. Oxygen and glucose deprivation/reperfusion (OGD/R) was established by culturing the cells in glucose-free DMEM and hypoxic conditions with 95%N_2_/5%CO_2_. After 2 h of hypoxia, the cells were transferred back to full culture medium under normal atmosphere and incubated for 24 h. At 3h before OGD/R, rIL-22 (100 ng/mL) was administrated. To knockdown JAK2 and STAT3 expression, PC12 cells were transfected with si-JAK2 and si-STAT3, respectively, using Lipofectamine 2000 according to the manufacturer's recommendation.

### 2.6. ELISA

After 24 h of reperfusion, blood specimens were obtained from mice and centrifuged to separate the serum. The levels of IL-1*β*, monocyte chemotactic protein- (MCP-) 1, and tumor necrosis factor- (TNF-) *α* were measured by ELISA kits (R&D Systems, USA) according to the manufacturer's instructions.

### 2.7. Oxidative Stress Detection

After 24 h of reperfusion, the brain tissues were rapidly removed and prepared as homogenates, and then, the supernatants were collected. For the cells, PC12 cells were harvested and lysed, and then, the supernatants were collected. The activity of total superoxide dismutase (SOD) and glutathione (GSH) and the concentration of malondialdehyde (MDA) were measured by commercial assay kits (Beyotime Biotechnology, China) according to the manufacturer's instructions.

### 2.8. Apoptosis Assay

Cell apoptosis was measured by a terminal deoxynucleotidyl transferase-mediated dUTP nick end labeling (TUNEL) assay kit, as previously described [31]. Briefly, the slices were incubated with TUNEL reagents, and DAPI solution was prepared according to the manufacturer's instructions. The number and ratio of TUNEL-positive cells were calculated based on the apoptosis index evaluated by an investigator blinded to the experiment.

### 2.9. Quantitative Real-Time RT-PCR

Total RNA was extracted from the ischemic hemisphere and PC12 cells using a TRIzol reagent and then reverse transcribed into cDNA according to the manufacturer's protocol. Real-time PCR analysis was performed using a LightCycler 480 qPCR System. The relative expression of target genes was normalized against *β*-actin mRNA. The primer sequences are presented in Table 1.

### 2.10. Western Blotting

Protein was extracted from the ischemic hemisphere and PC12 cells and then separated using SDS-PAGE. The proteins were transferred onto an Immobilon-P membrane (Millipore, USA). The membranes were incubated with primary antibodies against Bax, Bcl-2, p-JAK2, JAK2, p-STAT3, STAT3, and *β*-actin, followed by incubation with the secondary antibody. Finally, proteins on the membranes were detected using an Odyssey infrared imaging system (LI-COR, USA), and the protein expression levels were normalized to that of *β*-actin.

### 2.11. Statistical Analysis

Statistical analyses were performed using SPSS software. Normally distributed data are expressed as the mean ± standard deviation (SD). One-way analysis of variance (ANOVA) was used for comparisons among multiple groups, and when the differences were statistically significant, a post hoc Tukey test was carried out. Nonnormally distributed data are expressed as the median and quartiles. The Kruskal-Wallis *H* test was used for comparisons among multiple groups, and when the differences were statistically significant, the Mann-Whitney *U* test was carried out followed by Bonferroni correction. The Bonferroni correction is *α*′ = 0.05/*K*, where *K* is the number of comparisons. *P* values less than 0.05 were considered statistically significant.

## 3. Results

### 3.1. IL-22 Treatment Ameliorated Cerebral I/R Injury

**Figure 1:**
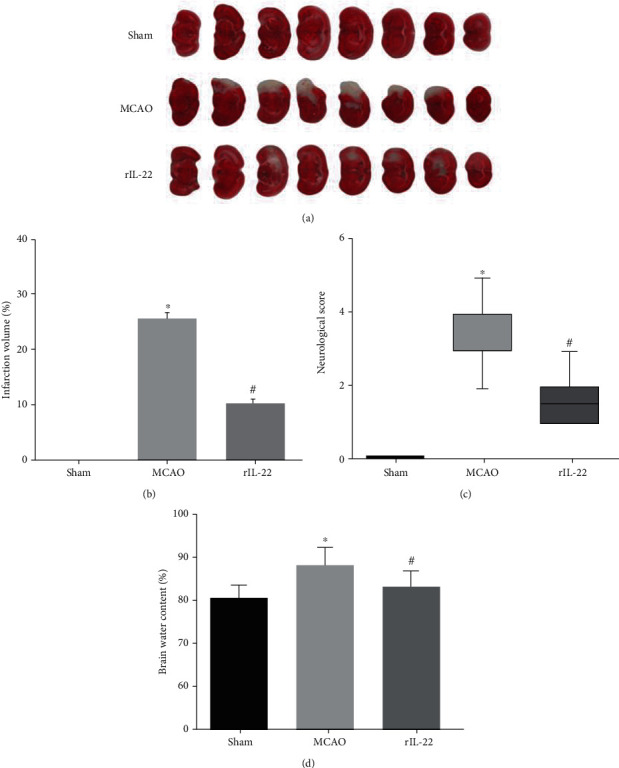
IL-22 treatment ameliorated cerebral I/R injury. (a) Representative sections of TTC staining in each group (*n* = 6). (b) Quantification of infarct volume in each group (*n* = 6). (c) Neurological deficits were assessed in each group (*n* = 8). (d) Brain water content was calculated in each group (*n* = 6). ^∗^*P* < 0.05 vs sham group; ^#^*P* < 0.05 vs MCAO group.

After ischemia-reperfusion, significant cerebral infarction was observed in the MCAO group, but IL-22 treatment significantly decreased the infarct volume of mice (Figures 1(a) and 1(b)). In addition, IL-22 administration significantly ameliorated neurological deficits and brain water content after cerebral I/R injury (Figures 1(c) and 1(d)).

### 3.2. IL-22 Treatment Inhibited the Inflammatory Response after Cerebral I/R Injury

**Figure 2:**
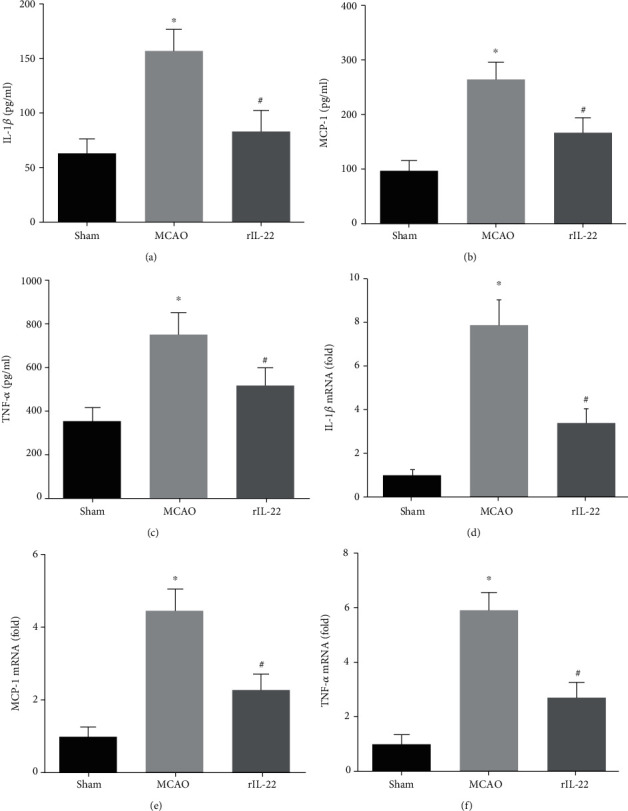
IL-22 treatment inhibited the inflammatory response after cerebral I/R injury. Serum levels of IL-1*β* (a), MCP-1 (b), and TNF-*α* (c) were measured by ELISA (*n* = 8). The mRNA expression of IL-1*β* (d), MCP-1 (e), and TNF-*α* (f) was detected in brain tissues (*n* = 8). ^∗^*P* < 0.05 vs. sham group; ^#^*P* < 0.05 vs. MCAO group.

Compared with the sham group, serum levels of inflammatory cytokines, including IL-1*β*, MCP-1, and TNF-*α*, in the MCAO group were significantly increased, while IL-22 treatment reduced the serum levels of these cytokines (Figures 2(a)–2(c)). Furthermore, IL-22 treatment also decreased the mRNA expression of IL-1*β*, MCP-1, and TNF-*α* in the ischemic cerebral cortex (Figures 2(d)–2(f)).

### 3.3. IL-22 Treatment Attenuated Oxidative Stress and Neuronal Apoptosis after Cerebral I/R Injury

**Figure 3:**
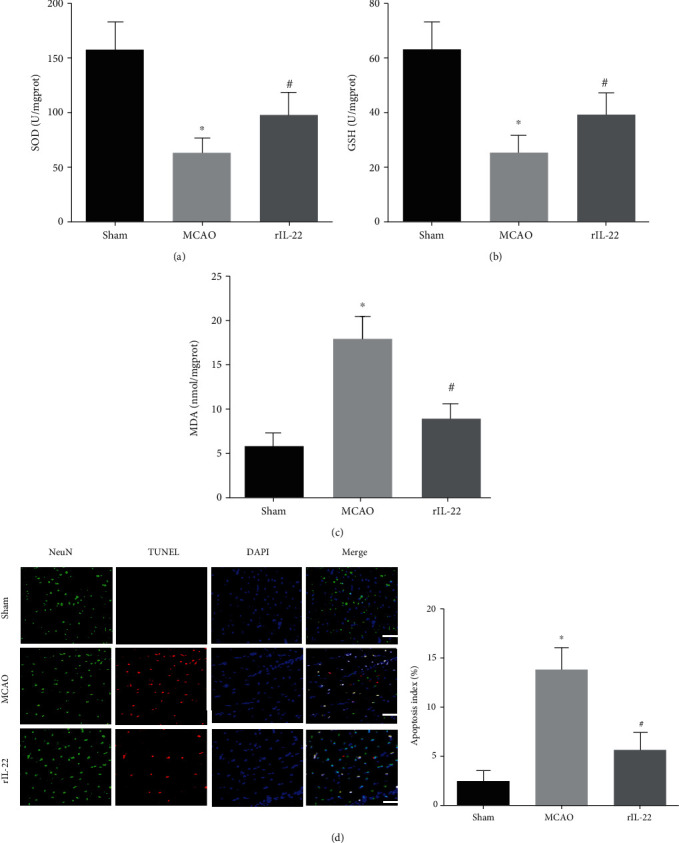
IL-22 treatment attenuated oxidative stress and neuronal apoptosis after cerebral I/R injury. The levels of SOD (a), GSH (b), and MDA (c) were detected in brain tissues (*n* = 6). (d) Neuronal apoptosis was detected by TUNEL staining combined with immunostaining for NeuN (*n* = 5, scale bar = 75 *μ*m). ^∗^*P* < 0.05 vs. sham group; ^#^*P* < 0.05 vs. MCAO group.

Compared with the sham group, the activities of SOD and GSH in the MCAO group were significantly decreased and the levels of MDA were significantly increased after cerebral I/R injury (Figures 3(a)–3(c)). Interestingly, the activities of SOD and GSH in the brain tissues were significantly increased, and the levels of MDA were significantly reduced in the rIL-22 group compared with those in the MCAO group (Figures 3(a)–3(c)). The TUNEL staining results also showed that IL-22 treatment significantly decreased neuronal apoptosis after cerebral I/R (Figure 3(d)).

### 3.4. IL-22 Treatment Inhibited OGD/R-Induced Inflammation and Oxidative Stress

**Figure 4:**
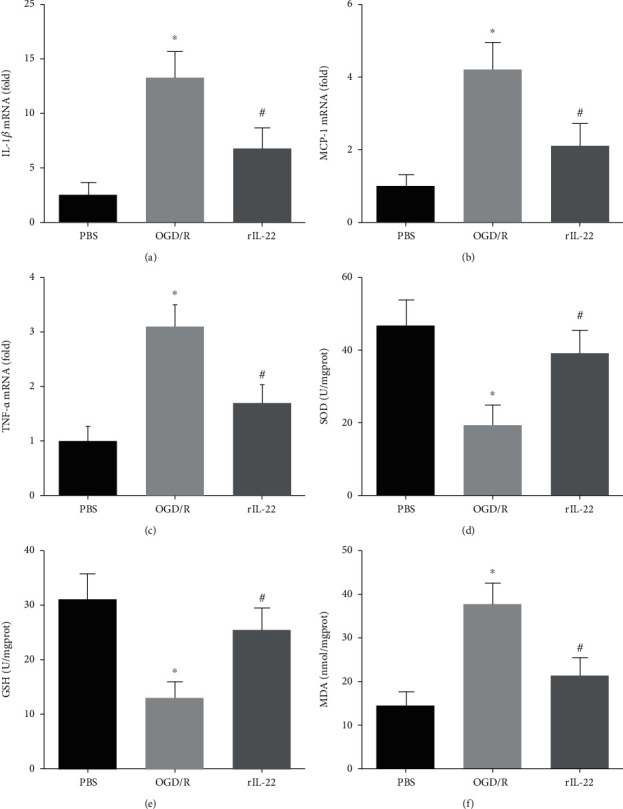
IL-22 treatment inhibited OGD/R-induced inflammation and oxidative stress. The mRNA expression of IL-1*β* (a), MCP-1 (b), and TNF-*α* (c) was detected in PC12 cells (*n* = 6). The levels of SOD (d), GSH (e), and MDA (f) were detected in PC12 cells (*n* = 6). ^∗^*P* < 0.05 vs. PBS group; ^#^*P* < 0.05 vs. OGD/R group.

Our results showed that IL-22 treatment significantly decreased the mRNA expression of IL-1*β*, MCP-1, and TNF-*α* after OGD/R (Figures 4(a)–4(c)). In addition, IL-22 treatment significantly increased the activities of SOD and GSH and reduced the levels of MDA compared with the OGD/R group (Figures 4(d)–4(f)).

### 3.5. IL-22 Treatment Attenuated OGD/R-Induced Neuronal Apoptosis

**Figure 5:**
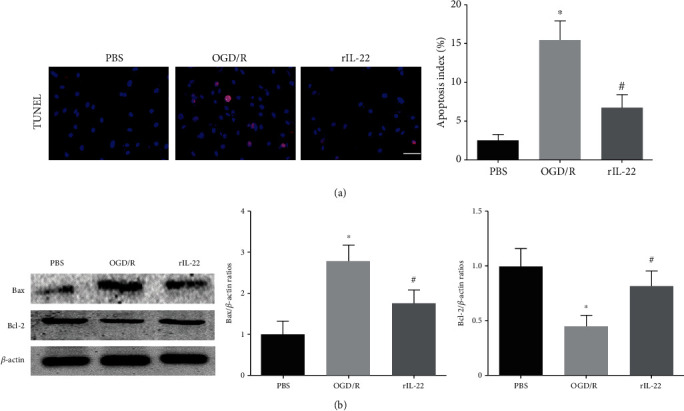
IL-22 treatment attenuated OGD/R-induced neuronal apoptosis. (a) Neuronal apoptosis was detected by TUNEL staining (*n* = 5, scale bar = 50 *μ*m). (b) The expression of Bax, Bcl-2, and *β*-actin was detected by western blotting (*n* = 4). ^∗^*P* < 0.05 vs. PBS group; ^#^*P* < 0.05 vs. OGD/R group.

The TUNEL staining results showed that the apoptotic index in the OGD/R group was significantly lower than that in the PBS group, while IL-22 treatment significantly diminished OGD/R-induced cell apoptosis (Figure 5(a)). In addition, IL-22 treatment significantly attenuated the restored Bcl-2 expression and reduced Bax expression after OGD/R insult (Figure 5(b)).

### 3.6. IL-22 Treatment Activated the JAK2/STAT3 Signaling Pathway

**Figure 6:**
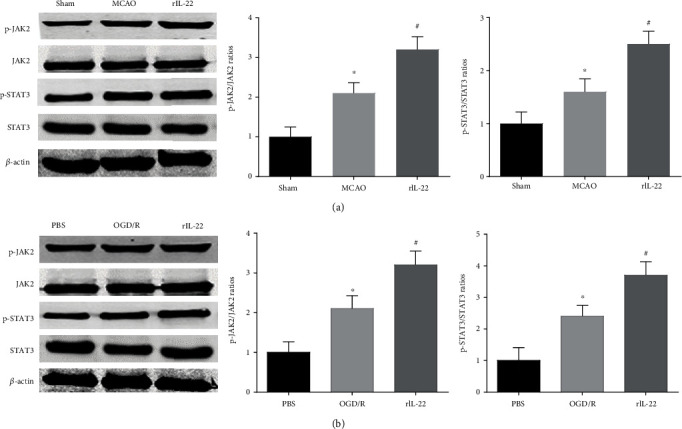
IL-22 treatment activated the JAK2/STAT3 signaling pathway. (a) The expression of p-JAK2, JAK2, p-STAT3, STAT3, and *β*-actin in the brain was detected by western blotting (*n* = 4). (b) The expression of p-JAK2, JAK2, p-STAT3, STAT3, and *β*-actin in PC12 cells was detected by western blotting (*n* = 4). ^∗^*P* < 0.05 vs. the sham or PBS group; ^#^*P* < 0.05 vs. the MCAO or OGD/R group.

Our results showed that the phosphorylation levels of JAK2 and STAT3 in the MCAO group were significantly higher than those in the sham group, and IL-22 treatment further increased JAK2 and STAT3 phosphorylation levels (Figure 6(a)). In addition, our results also showed that IL-22 treatment upregulated JAK2 and STAT3 phosphorylation levels in PC12 cells after OGD/R insult (Figure 6(b)).

### 3.7. JAK2 and STAT3 Knockdown Abolished IL-22-Mediated Neuroprotection

**Figure 7:**
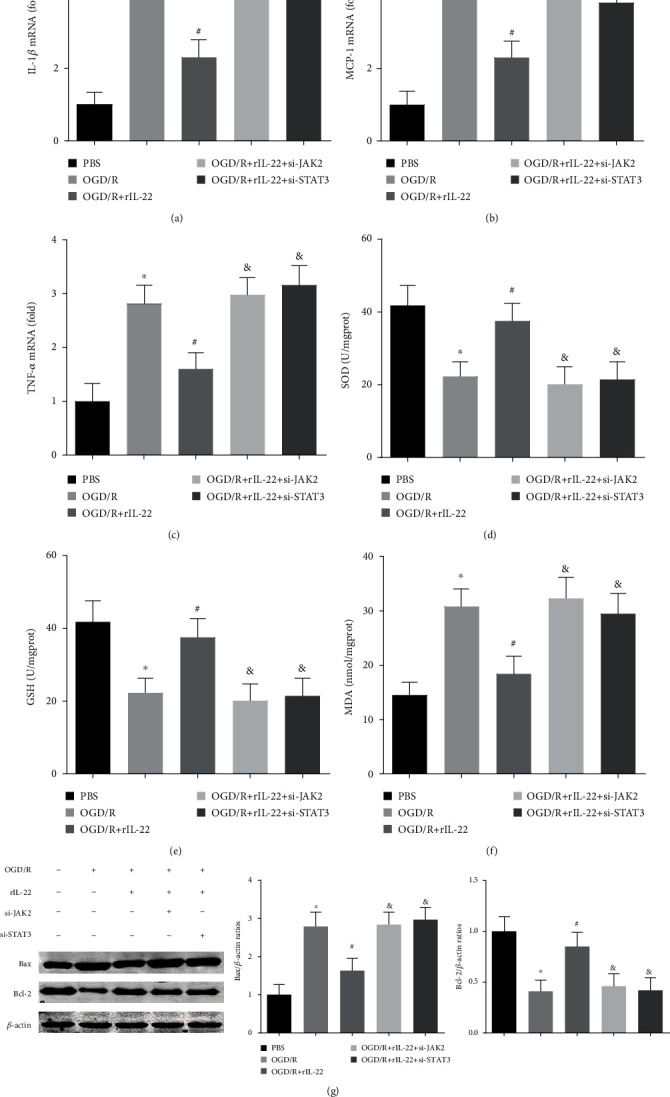
JAK2 and STAT3 knockdown abolished IL-22-mediated neuroprotection. The mRNA expression of IL-1*β* (a), MCP-1 (b), and TNF-*α* (c) was detected in PC12 cells (*n* = 6). The levels of SOD (d), GSH (e), and MDA (f) were detected in PC12 cells (*n* = 6). (g) The expression of Bax, Bcl-2, and *β*-actin was detected by western blotting (*n* = 4). ^∗^*P* < 0.05 vs. PBS group; ^#^*P* < 0.05 vs. OGD/R group; ^&^*P* < 0.05 vs. OGD/R+rIL-22 group.

To further confirm the effect of the JAK2/STAT3 signaling pathway in IL-22-mediated neuroprotection, transfection with si-JAK2 and si-STAT3 was performed to knock down JAK2 and STAT3 expression in vitro, respectively. The results showed that JAK2 and STAT3 knockdown abolished the IL-22-mediated anti-inflammatory effects by increasing IL-1*β*, MCP-1, and TNF-*α* mRNA expression (Figures 7(a)–7(c)). In addition, JAK2 and STAT3 knockdown also attenuated IL-22-mediated antioxidative stress and antiapoptotic effects (Figures 7(d)–7(g)). The above results revealed that the JAK2/STAT3 pathway plays a central role in IL-22-mediated neuroprotective effects.

## 4. Discussion

In the current study, we investigated the protective effect of IL-22 against cerebral I/R injury. We provided direct evidence that IL-22 treatment decreased infarct size, neurological deficits, and brain water content in mice subjected to cerebral I/R injury. IL-22 treatment remarkably attenuated the inflammatory response, oxidative stress, and neuronal apoptosis after cerebral I/R injury. In addition, IL-22 treatment decreased the inflammatory response, oxidative stress, and apoptosis of PC12 cells after OGD/R insult. Moreover, IL-22 treatment significantly increased JAK2 and STAT3 phosphorylation levels in mice and PC12 cells, and STAT3 knockdown abolished the IL-22-mediated neuroprotective function. These findings suggest that IL-22 could be exploited as a potential therapeutic agent for ischemic stroke and cerebral I/R injury.

Based on similarities in structure and receptor subunits, the IL-10 family comprises six members, including IL-10, IL-19, IL-20, IL-22, IL-24, and IL-26 [32–34]. As an anti-inflammatory cytokine, the neuroprotective effect of IL-10 on cerebral I/R injury has been identified in numerous studies [35, 36]. In addition, IL-19 administration also reduced ischemia-induced brain infarct and neurological deficits in mice after experimental ischemic stroke, indicating that IL-19 is a novel therapeutic target for cerebral I/R injury [37]. However, IL-20 expression was upregulated in the serum and brain tissue of rats after cerebral I/R, and anti-IL-20 neutralizing antibody administration ameliorated MCAO-induced brain infarction in rats [38]. IL-22 was first identified as a product of CD4+ T cell subsets, and subsequent studies demonstrated that IL-22 is also secreted by macrophage, natural killer cells, and natural killer T cells [24, 39]. Previous studies reported that IL-22 was expressed in human brain tissue and mouse brain, and IL-22 treatment protected nutriment-deprived astrocytes from cell death [40, 41]. Liu et al. also reported that IL-22 treatment significantly inhibited serum starvation-induced PC12 cell death, indicating that IL-22 may confer a neuroprotective function [42].

The results showed that rIL-22 administered in advance significantly decreased the infarct volume and ameliorated neurological deficits and brain water content after cerebral I/R injury in mice. In addition, IL-22 treatment also diminished OGD/R-induced neuronal injury and apoptosis in vitro. These findings demonstrated that IL-22 exerts a neuroprotective effect on cerebral ischemic injury.

The inflammatory response plays a pivotal role in the pathophysiology of I/R-induced cerebral injury. After stroke, the interruption and reperfusion of blood flow in brain tissue trigger inflammatory cell infiltration and cause a robust inflammatory response, which induces neuronal apoptosis and death [43, 44]. Multiple inflammatory-related cytokines are released in ischemic brain injury and participate in the damage and repair process of brain tissue, including ILs, TNF, interferon, and chemokines [45, 46]. In addition, numerous data suggest that the inflammatory response is closely related to oxidative stress and aggravating I/R-induced cerebral injury [47, 48]. Thus, it is clear that therapeutic drugs targeting the inflammatory response and oxidative stress can be very effective in improving cerebral I/R injury. Previous research has shown that IL-22 is an inflammation-related cytokine and has anti-inflammatory and antioxidative stress effects. Thus, we investigated whether IL-22 affects the inflammatory response and oxidative stress in MCAO-induced cerebral I/R injury.

Our results showed that serum levels of inflammatory cytokines, including IL-1*β*, MCP-1, and TNF-*α*, in the MCAO group were significantly higher than those in the sham group, while IL-22 treatment significantly reduced the serum levels of these cytokines. In addition, IL-22 treatment also decreased the mRNA expression of IL-1*β*, MCP-1, and TNF-*α* in brain tissue after cerebral I/R injury. To assess the effects of IL-22 on cerebral I/R-induced oxidative stress, we measured the SOD and GSH activities and MDA contents in brain tissues. The results showed that the activities of SOD and GSH in the brain tissues were significantly increased and the levels of MDA were significantly reduced in the rIL-22 group compared with those in the MCAO group.

Signaling through the JAK/STAT pathway is important for the progression of neurological diseases, including stroke, traumatic brain injury, status epilepticus, brain tumors, and neurodegenerative diseases [49, 50]. Many lines of evidence have indicated that JAK2/STAT3 signaling is activated in the early stage of cerebral ischemia and mediates oxidative stress, the inflammatory response, and neuronal apoptosis [49, 51]. Kinouchi et al. reported that pioglitazone protects against cerebral I/R injury by activating the JAK2/STAT3 signaling pathway [52]. Liu et al. reported that diosmin inhibits neuronal apoptosis by activating the JAK2/STAT3 signaling pathway after cerebral ischemia in mice [53]. Accumulating evidence suggests that JAK2/STAT3 is a major downstream signal of IL-22 and mediates its hepatoprotective and cardioprotective functions [25, 39].

In the current study, we investigated the role of IL-22 treatment in JAK2/STAT3 signaling after cerebral I/R. The results showed that the phosphorylation levels of JAK2 and STAT3 were upregulated in mice after cerebral I/R injury and in PC12 cells following OGD/R. In addition, IL-22 treatment further increased JAK2 and STAT3 phosphorylation levels, and STAT3 knockdown abolished the IL-22-mediated neuroprotective function. These findings indicate that the neuroprotective actions of IL-22 are related to the JAK2/STAT3 signaling pathway.

Over the last decade, evidence supporting combination therapies has been obtained from a variety of studies in many types of animal models [54, 55]. The combination of thrombolysis and neuroprotection has been considered a promising approach for the treatment of acute ischemic stroke [55, 56]. Tissue plasminogen activator (tPA) is the only treatment approved by the USA FDA for acute ischemic stroke; it dissolves the obstructive clot to restore cerebral blood flow [57]. Neuroprotective agents attenuate the inflammatory response and suppress molecules that mediate thrombosis and blood-brain barrier disruption induced by ischemia such that the benefits of tPA may be extended [55, 56]. Our study shows that IL-22 may be a promising neuroprotective agent; however, the combined effect of IL-22 and tPA is still unclear and will be the focus of our next study.

In conclusion, our findings provide preliminary evidence demonstrating the roles of IL-22 in cerebral I/R injury. IL-22 treatment prevented I/R-induced cerebral injury and neurological deficits by alleviating the inflammatory response, oxidative stress, and neuronal apoptosis. Our data indicate that IL-22 may serve as an attractive therapeutic target for treating ischemic stroke and cerebral I/R injury.

## Figures and Tables

**Table 1:** Primer sequences for RT-PCR assays.

Gene	Species	Sequence (5′-3′)	
IL-1*β*	Mouse	Forward	GGGCCTCAAAGGAAAGAATC
		Reverse	TACCAGTTGGGGAACTCTGC
IL-1*β*	Rat	Forward	GTGCTGTCTGACCCATGTGA
		Reverse	CACAGGGATTTTGTCGTTGCT
MCP-1	Mouse	Forward	GAGGTCACTCCTATCCTCTGG
		Reverse	GCCATTTCCTCCGACTTTTCTC
MCP-1	Rat	Forward	AGCATCCACGTGCTGTCTC
		Reverse	GATCATCTTGCCAGTGAATGAG
TNF-*α*	Mouse	Forward	CCCAGGGACCTCTCTCTAATC
		Reverse	ATGGGCTACAGGCTTGTCACT
TNF-*α*	Rat	Forward	CTACTCCCAGGTTCTCTTCAA
		Reverse	GCTGACTTTCTCCTGGTATGA
*β*-Actin	Mouse	Forward	TATTGGCAACGAGCGGTTCC
		Reverse	GGCATAGAGGTCTTTACGGATGT
*β*-Actin	Rat	Forward	CAAGAAGGTGGTGAAGCAG
		Reverse	AAAGGTGGAAGAATGGGAG

## Data Availability

The datasets generated and/or analyzed during the current study are available from the corresponding author on reasonable request in compliance with ethical standards.
